# Spondyloepiphyseal Dysplasia Congenita: A Rare Cause of Respiratory Distress

**DOI:** 10.7759/cureus.5101

**Published:** 2019-07-08

**Authors:** Sidra Saleem, Arsalan Anwar, Pulwasha M Iftikhar, Zauraiz Anjum, Zemal Tariq

**Affiliations:** 1 Neurology, University of Toledo, Toledo, USA; 2 Neurology, University Hospitals Cleveland Medical Center, Cleveland, USA; 3 Obstetrics and Gynecology, St. John's University, New York, USA; 4 Internal Medicine, Fatima Jinnah Medical University, Lahore, PAK; 5 Internal Medicine, Gujranwala Medical College, Gujranwala, PAK

**Keywords:** skeletal dysplasia, spondyloepiphyseal dysplasia congenita, collagen, respiratory distress, ossification

## Abstract

Spondyloepiphysal dysplasia (SED) is an inheritable dysplasia of the bone due to a defect in collagen. It has a prevalence of 3.4 per million. It has two important types, congenita and tarda, which are differentiated by the age presentation and heritage mode. SED congenita can present a significant reduction in the upper segment to a lower segment ratio. Collagen mutation results in abnormal growth and development of spine and limb bones. The complex pattern of craniofacial anomalies is due to defective ossification and connective tissue problem. We here present the case of a three-hour-old girl with a short trunk and craniofacial anomalies that brought in respiratory distress to the neonatal intensive care unit. This condition is rare and thus poses a major diagnostic challenge at an early stage.

## Introduction

Spondyloepiphyseal dysplasia (SED), an inheritable bone dysplasia, has two types, SED tarda and congenita [1]. In 1966, Spranger first described spondyloepiphyseal dysplasia congenita (SEDC) and was later explained in 1970 as a “heritable dysplasia manifested at birth with the smallness of stature and retarded ossification of the vertebral bodies, extremities and pelvis” [2]. According to the National Institute of Health (NIH) data, 175 cases are reported in the literature so far [3]. Growth deficiency usually occurs before birth and goes on throughout life. Short stature is disproportionate as the arm is slightly longer than the torso. Other clinical manifestations include the flat face, cleft palate, barrel-shaped chest, muscle hypotonia, clubfoot, myopia and sensorineural deafness as the primary defect is in type II collagen synthesis. It is an autosomal dominant but many patients acquire the disease because of a new mutation.

As age progresses, vertebrae become irregular and flattened, resulting in kyphoscoliosis, this increases the risk of cervical myelopathy. It can also cause limb shortening, pelvic dysplasia, and diminishing ossification in the femoral head with coxa vara. In the elbow, knee and hip joint, decreased joint mobility and stiffness are present. Hip joint involvement can lead to coxa vara as thigh bones are angled to the body, knee joint participation can lead to genu varum and valgum. Secondary complications include atlantoaxial instability, recurrent otitis media, retinal detachment and myopia, lumbar lordosis, sciatica, and delayed motor development [4]. Some infants have breathing problems due to an underdeveloped and small ribcage. We here present the case of a 3-hour-old neonate with breathing difficulty and weak cry at birth. Upon clinical examination a short trunk and dysmorphic features were noted that led to further skeletal survey and genetic testing.

## Case presentation

**Figure 1:**
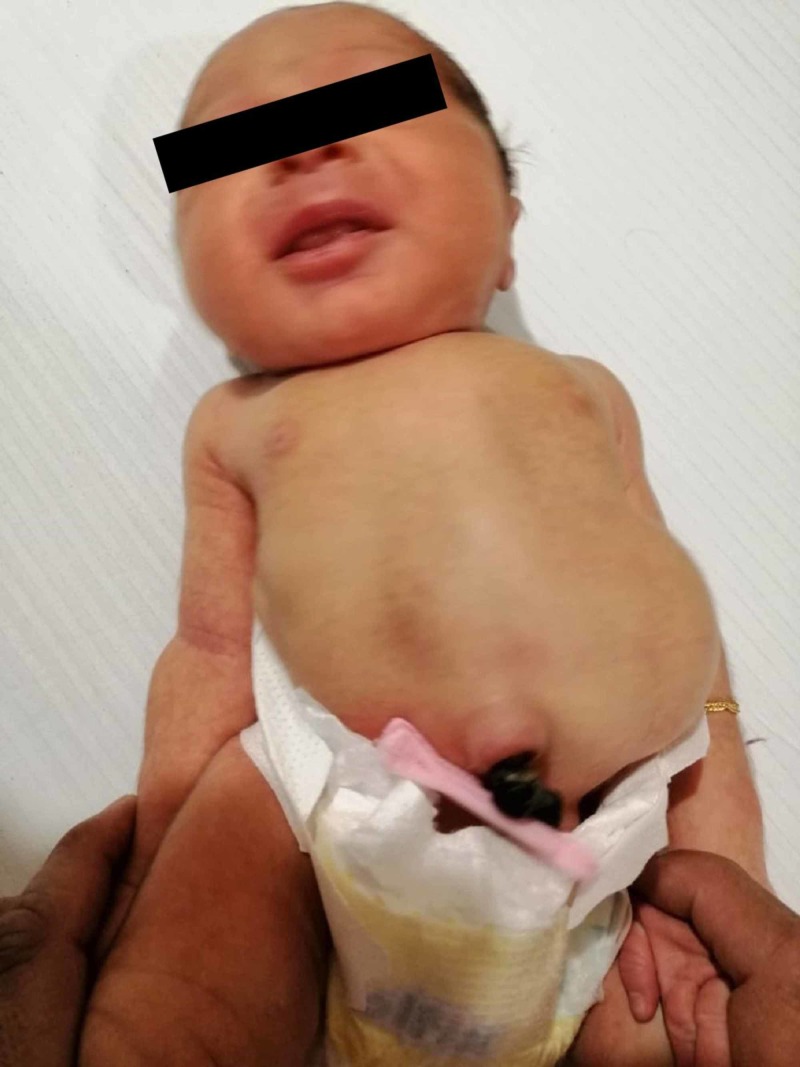
7-day-old neonate, short trunk compared with upper limbs and blood stained umbilicus

A 3-hour-old term female was born to a non-consanguinity married couple. She was brought to NICU, with complaints of shortness of breath, tachypnea, grunting, a weak cry, diminished muscle tone, and short trunk. Her mother was a booked patient, she had regular antenatal checkups and scans, and the pregnancy was uneventful. On clinical examination of the patient, her temperature was 98.6°F, pulse 140/min, respiratory rate was 85/minute and oxygen saturation was 85%. She had dysmorphic characteristics, short trunk, and disproportionate limbs. Her Moro's and grasping reflex were normal but sucking reflex was weak, and there was no rooting reflex. Her head was large compared to the trunk (Figures 1); her face was flat with small mandible and retrognathia. Her head sutures with large clavirum were wide open and the neck was short. However, the spine and limbs were well aligned, but the chest was wide and short. According to anthropometric measurements, her body length was 40 cm, well below the 10th percentile, with disproportionate upper body segment to lower body segment ratio 1.3. The reference range of upper to lower body segment ratio is 1.7 at birth. Her weight was 2600 grams and head circumference was 34 cm; these were within the lower normal ranges. On auscultation of the chest, harsh vesicular breathing was present. Rest of the examination was normal. We kept the child in incubator and oxygen by continous positive airway pressure (CPAP).

**Figure 2:**
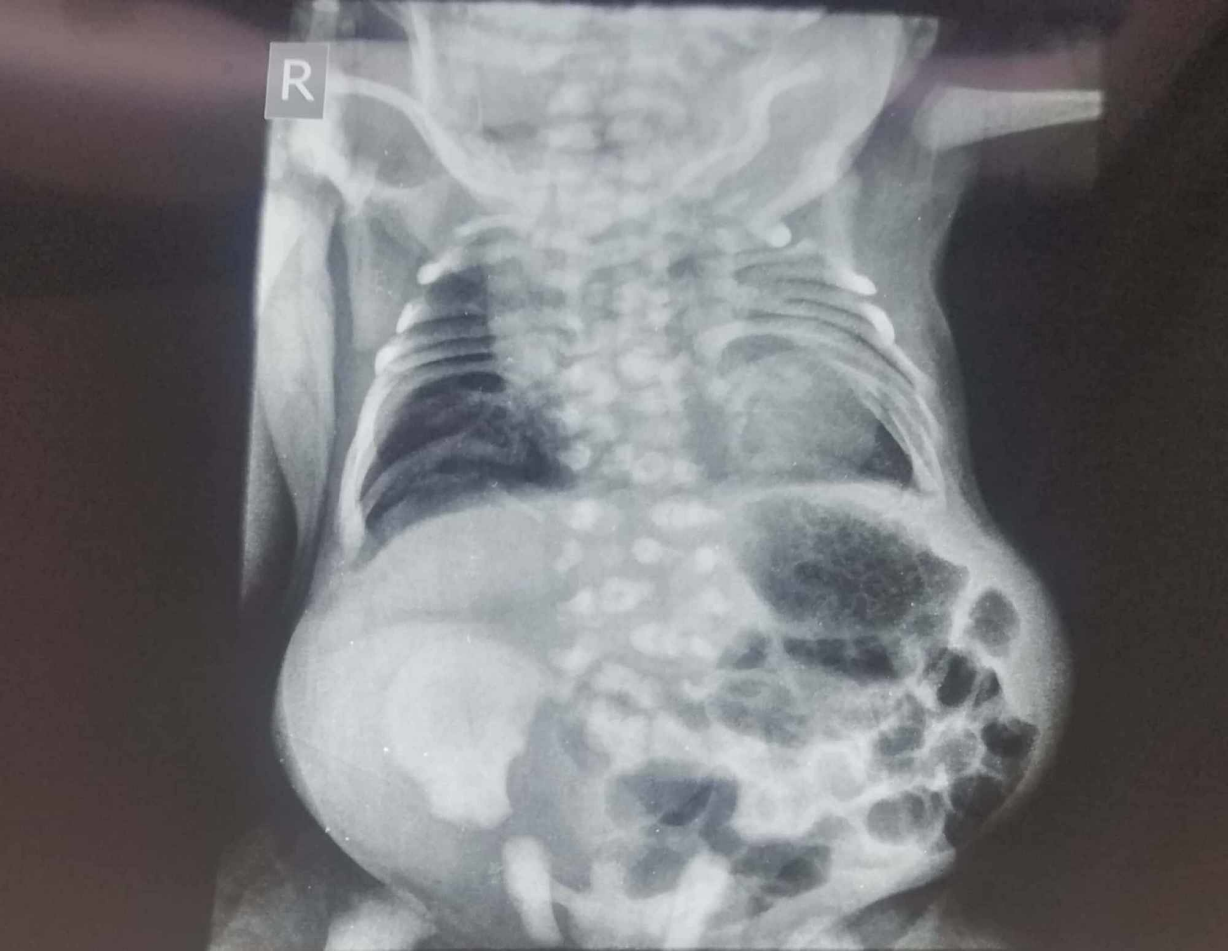
Radiology showing dysgenesis of vertebrae and pelvic bones

**Figure 3:**
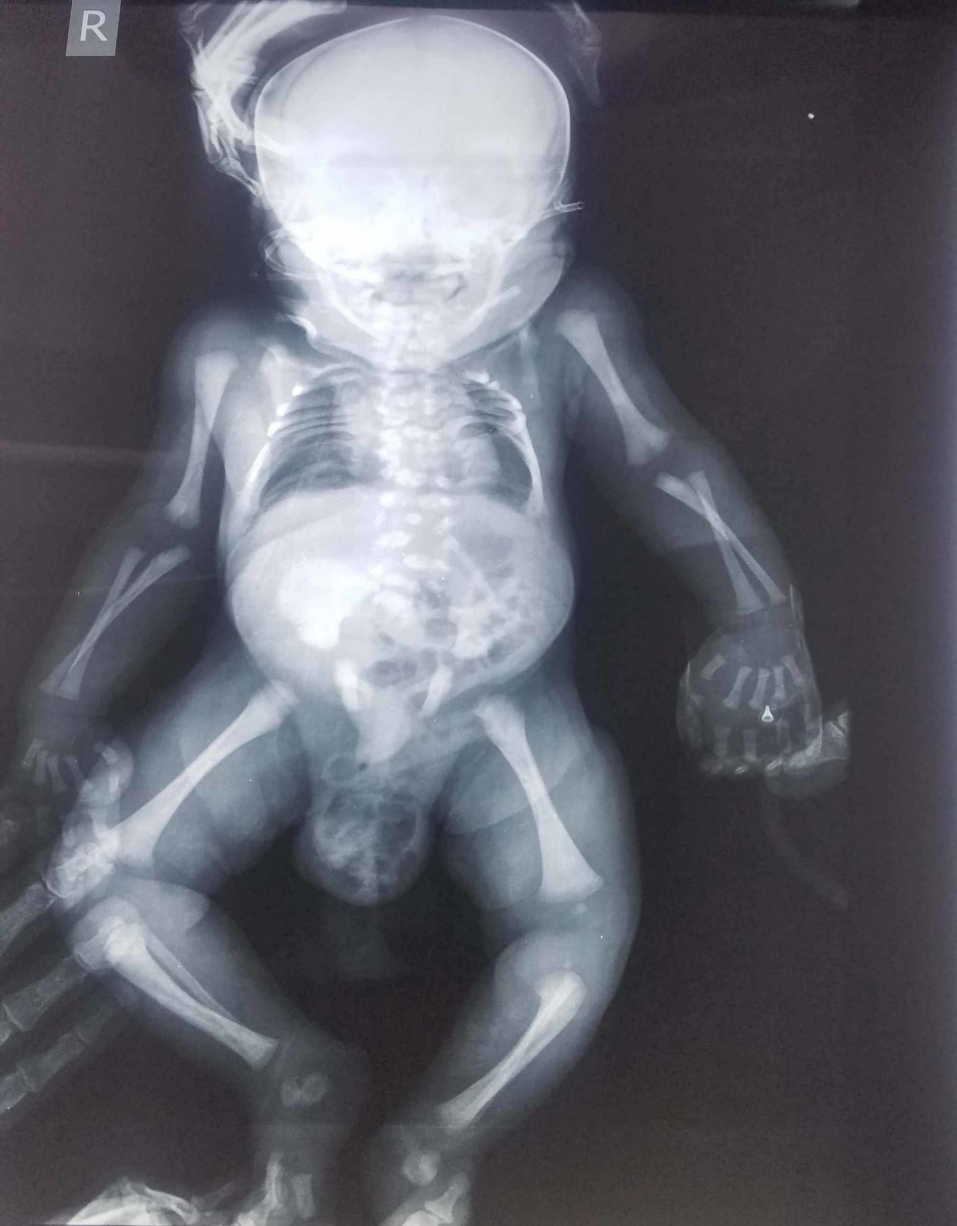
Radiology showing decrease ossification of bone

On further assessment and imaging, the complete skeletal survey showed widening of interpedicular distance with few hemi and butterfly vertebrae noted in the lower dorsal and lumbar spine, partial sacral dysgenesis, thoracic cage deformation with rib crowding in the upper half (Figures 2, 3).

Abdominal ultrasound and echocardiography were normal. All blood markers and metabolic profiles were normal. Exome sequencing of DNA was performed, which showed heterozygous mutation of the COL2A1 gene. The diagnosis was confirmed by clinical, radiological and genetic characteristics of SEDC with the decreased upper segment to lower segment ratio and craniofacial structures anomalies like flat facies, large clavirum, retrognathia, and barrel-shaped chest. After five days on CPAP, the child's oxygen saturation was improved to 95%. She was discharged with advice to parents to follow up with the pediatrician, orthopedic surgeons, spine specialist, ophthalmologists, physical therapists, and genetic counselor.

## Discussion

Skeletal dysplasia is a heterogeneous group of bone growth disorder, resulting in short stature, skeletal abnormalities and with some hearing and vision difficulties [5]. Over 450 different entities have been described on the basis of radiological, molecular and biochemical criteria [6]. SED is one of the rare disorders that come under the umbrella of skeletal dysplasia. It has two types according to the mode of inheritance and age at presentation: spondyloepiphyseal dysplasiatarda (SEDT), and spondyloepiphyseal dysplasiacongenital (SEDC). Incidence of SEDC is 1:100,000 per year and prevalence is 3.4 per million. It is caused by the mutation of type II collagen (COL2A1) gene which is located within the triple helical domain of the protein. This results in an abnormal collagen protein synthesis, which is an important cartilage component [7]. More than 27 mutations have been identified, but the genotype and phenotype relation is unclear. By identifying the novel mutation, we can identify this relation that can help us in better understanding of pathogenesis [8]. Asymmetric growth presents in the femoral epiphysis and causes genu valgum. Atlantoaxial instability is present due to abnormal ossification of the tip of the odontoid process. Myopia is due to defective type II collagen in the neural retina, optic vesicle, sclera of the eye and conjunctival epithelium. Ear problems are due to otic vesicle epithelium and cartilaginous eustachian tube [9]. Many neonates present with respiratory problems soon after birth due to the small thorax with decreased intra-thoracic volume. However, the other causes of respiratory difficulty are abnormal chest wall compliance, compressed medulla and upper cervical spine instability, tracheomalacia and horizontal rib alignment resulting in decreased tidal volume [10].

Al Kaissi et al. in their case series present clinical presentation of SEDC as short stature, short neck, wide frontal area, wide-set ears, full cheeks, and a barrel-shaped chest. This is the same as the clinical presentation of our case [11]. The patient in this study meets SEDC's diagnostic criteria and Al Kaissi et al. concluded that in the differential diagnosis of the short trunk and skeletal dysplasia, SEDC should always be considered to avoid unnecessary workup. The study conducted by Huang et al. in Chinese families showed short trunk dwarfism was noted at birth and radiographic findings of ovoid vertebral bodies, flattening of the acetabular roof, and shortening of the femoral neck. Similarly, in our patient, there was a disproportion in the upper body segment and lower body segment [12]. This rare disease is of major clinical importance in order to prevent unnecessary interventions and it could be serving a tool for the proband. The diagnosis of SEDC may be facilitated by the careful clinical evaluation of newborn, radiological findings of dysplastic bones, especially vertebrae, echocardiography, abdominal ultrasound and feeding evaluation to rule out VACTERL (vertebral defects, anal atresia, cardiac defects, tracheo-esophageal fistula, renal anomalies, and limb abnormalities) [13]. COL2A1 plays a major role in the diagnosis of SEDC.

Jurgens et al. described COL2A1 heterozygous mutation at chromosome 12q after genetic testing due to glycine to serine substitution. This mutation interferes with type II collagen assembly in the pro-alpha1 (II) chain and may involve more than one subunit of procollagen in collagen homotrimers [14]. This mutation was present in our case as well and it was combined with clinical and radiological testing to confirm the diagnosis.

## Conclusions

This case provides insight into the variable presentation of SEDC at an early age as a novel mutation for clinical intervention and genetic counseling. In order to better comprehend COL2A1 pathology, we need more comprehensive research of skeletal dysplasia in big populations and ethnic diversities. It will also assist to supplement SEDC's knowledge of the connection between genotype and phenotype.
